# Mosquito swarm counting via attention-based multi-scale convolutional neural network

**DOI:** 10.1038/s41598-023-30387-4

**Published:** 2023-03-14

**Authors:** Huahua Chen, Junhao Ren, Wensheng Sun, Juan Hou, Ziping Miao

**Affiliations:** 1 School of Communication Engineering, Hangzhou Dianzi University, 1158 2nd Street, Hangzhou, China; 2 Zhejiang Provincial Center for Disease Control and Prevention, 630 Xincheng Road, Hangzhou, China

**Keywords:** Machine learning, Zoology, Entomology, Signal processing

## Abstract

Monitoring mosquito density to predict the risk of transmission of the virus and develop a response in advance is an important part of prevention efforts. This paper aims to estimate accurately the mosquito swarm count from a given image. To this end, we proposed an attention-based multi-scale mosquito swarm counting model that consists of the feature extraction network (FEN) and attention based multi-scale regression network (AMRN). The FEN uses VGG-16 network to extract low-level features of mosquitoes. The AMRN adopts a multi-scale convolutional neural network, and with a squeeze and excitation channel attention module in the branch with a 7 × 7 convolution kernel to extract high-level features, map the feature map to the mosquito swarm density map and estimate mosquitoes count. We collected and labelled a data set that includes 391 mosquito swarm images with 15,466 mosquitoes. Experiments show that our method performs well on the data set and achieves mean absolute error (MAE) of 1.810 and root mean square error (RMSE) of 3.467.

## Introduction

As carbon dioxide emissions rise and global warming intensifies, mosquitoes become more active, and the risk of contracting mosquito-borne diseases increases. Mosquito-borne infectious diseases transmit pathogens through mosquito bites. Current climate change, ecological change, globalization, urbanization and other factors, as well as macro policies around the world, will have an important impact on the epidemic of this disease^1^. It has become a global public health problem^2,3^. Surveillance and control of mosquitoes is an important way to stop the spread of mosquito-borne diseases. Scientific and effective mosquitoes surveillance can evaluate the risk of outbreaks to provide early warning and take the best mosquitoes measure to control. It also provides a scientific basis for handling mosquito-borne disease outbreaks and effectively prevents the further spread of the epidemic. However, the existing mosquito counting methods, such as the human landing catch method and labor hour method, mainly rely on manual counting^4,5^, which have a large workload and are low efficiency. In addition, operators are easy to be bitten, so there is a certain risk of infection, and it is not easy to count high-density mosquito swarm. So it is of great practical significance to study a fast automatic counting method with a low error rate.

## Related work

With the advancement of science and technology, many techniques have made great breakthroughs in the fields of computer vision and pattern recognition recently, and they play an important role in many applications of different fields. Mosquito swarm counting based on image processing is a non-contact technique with high efficiency and high accuracy, and it can greatly reduce labor costs. Many counting methods based on image processing have been developed. Existing counting methods can be classified into detection-based methods, segmentation-based methods, and regression-based methods.

### Detection-based methods

Traditional detection-based counting methods consist of steps including image acquisition, preprocessing, image feature extraction and refinement, pattern recognition, and counting^6,7^. The key step is feature extraction and refinement. Early research focused on extracting global features such as grayscale histogram^8^, color^9^, and texture of images^10^. Although such features are easy to extract, the representation based on global features does not work well in complex backgrounds. Therefore, some researchers extract various local features and fuse them as the final feature. However, these methods are not good for mosquito swarm counting especially when the mosquito swarm density is high and some mosquitoes touch each other.

### Segmentation-based methods

Segmentation-based counting methods segment objects from the background image and counts them^11,12^. These methods have achieved some good results. A limitation of these methods is that the segmentation results depend on image segmentation methods, which greatly affects the subsequent counting. Meanwhile the segmentation process is influenced by the image quality, adhesions between objects, isolated noise, etc., resulting in poor counting results.

### Regression-based methods

With the development of the research, regression-based methods provide a new solution to the counting problem. These methods can be classified into two classes, regression for objects counting and regression for objects density map. The main idea of regression for objects counting is to learn a mapping between features and the number of objects^13,14^. This class method typically works by segmenting the foreground, extracting various features from the foreground, and utilizing a regression function such as ridge regression or Gaussian process regression estimating the number of objects^15^. So foreground segmentation results have great influence on the subsequent counting. Regression for objects density map estimates the density map by a regression function. Unlike the former class method, this class method is to learn the mapping between the features in the local area and their density map and indirectly estimate the objects’ density map using algorithms such as random forest^16^, then obtain objects’ count by integrating based on the density map. With the development of deep learning, the methods of density map estimation based on convolutional neural networks (CNNs)^17,18^ have been gradually favored by researchers. The methods can output the density map whose integration is the number of objects when the network inputs the image, which simplifies the estimation steps while achieving a good performance.

Among the above counting methods there is no literature report on mosquito swarm counting. In this paper, we aim to perform accurate mosquito swarm counting from any still image, with any camera perspective and crowd density. It is a quite challenging task, since we need to overcome a number of challenges:

1. Foreground segmentation is essential and indispensable in most computer vision tasks. However, in our task, we have to estimate the mosquitoes count without segmenting the foreground because it requires pixel-by-pixel labelling and inaccurate segmentation results will have adverse effects on the subsequent counting, especially there is no definitive information about scene geometry or motion, mosquito swarm size in the dataset, and even the view point of the image can be arbitrary. It is almost impossible to accurately segment the mosquito swarm from its background.
2. Since the scale of mosquito can vary significantly in the images, it is difficult to hand-craft features for all different scales. We need to obtain features of different scales by automatic learning effective features to accurately estimate mosquito swarm count in different images.
3. The density and distribution of mosquito swarm in the data set are significantly different, and even there are occlusion and adhesion among mosquitoes in some images. In addition, fewer pixels of mosquitoes in the image makes the task more difficult.

To conquer the above challenges, we propose an attention-based multi-scale convolutional neural network (CNN) for mosquito swarm counting in any still image. It includes the feature extraction network (FEN) that consists of the first 10 layers of VGG-16 network and the attention based multi-scale regression network (AMRN) which consists of three parallel CNNs that have local receptive fields of different sizes. And a squeeze-and-excitation (SE) channel attention module is introduced into the AMRN to emphasize informative features. The output feature maps of all CNNs are concatenated and mapped to the density map whose integration is the mosquito swarm count. The main contributions of this paper are as follows:

1. Use the first 10 layers of VGG-16 network as the feature extraction network, which deepens the network and enables the subsequent ARMN to extract more complex high-level features of different sizes. It improves the density map accuracy of the network.
2. Multi-branch architecture is adopted in the AMRN to extract features of different scales. The three different kernel sizes in the three branches correspond to receptive fields of three different sizes so that the learned features in three branches of CNNs are adaptive to large variation in mosquito size and details due to different perspectives or image resolutions.
3. A channel attention module is introduced into the AMRN to increase the sensitivity of the network to the informative features and to increase the asymmetry of the AMRN structure to learn more different features, which helps the network to preserve the more information and improve the count estimation accuracy.
4. The built network has no restrictions on input image size. The input image to the model can be arbitrary size, and the output is an estimate of the density of the mosquito swarm from which a swarm count can be derived.
5. We build a new dataset for evaluation of mosquito swarm counting methods. Mosquito swarm datasets have not been reported in the literatures. In this work, we build a new mosquito swarm dataset of 391 mosquito swarm images with 14,566 accurately labeled mosquitoes. As far as we know, it is the first mosquito swarm counting dataset.

## Methods

In this paper, we estimate the density map by an attention-based multi-scale CNN. To solve the problems that effectively extract features of mosquitoes at different scales, we deploy the FEN to extract the low-level features and deepen the network, and the AMRN with an SE module to extract more complex high-level features of different scales.

### Network structure

**Figure 1:**
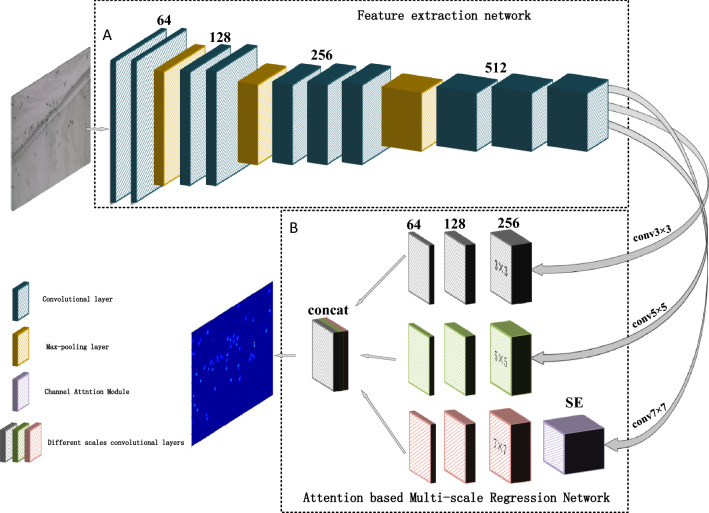
The framework of the proposed network. **(A**) The feature extraction network (FEN). (**B**) The attention based multi-scale regression network (AMRN). The input is a mosquito swarm image and the output is the corresponding density map.

Deep networks require massive training data. However, it is not feasible to collect so much data with their corresponding labels in mosquito swarm counting. So it is not a good way to train a deep network from scratch, which will easily lead to overfitting the network. It is a desirable method to use transfer learning between task domains^19^. The pre-trained model parameters from classification tasks are loaded into the proposed model, and then it is fine-tuned to convergence. The structure of the proposed network is shown in Fig. 1. It consists of parts A and B. They are the FEN and the AMRN respectively. The FEN consists of the first ten layers of VGG-16 network. The AMRN consists of three parallel CNNs that have local receptive fields of different sizes. Rectified linear unit (ReLU) is used as an activation function after each CNN. The output feature maps of all CNNs are concatenated and mapped to the density map. SE channel attention module is introduced into the AMRN to assign weights to feature maps according to their importance so that the feature representation ability of regions where mosquitoes stay can be boosted. The FEN loads the pre-trained model to extract low-level features, and the AMRN extracts more complex high-level features of different scales and then estimates the density map.

### Feature extraction network (FEN)

Borrowed from the literatures^18,20–22^, VGG-16 network is used to build the FEN by comparing different pre-trained model structures. We removed the fully connected layers of VGG-16 network to make the model adaptable to variable size inputs. VGG-16 model has 5 max pooling layers, and its output resolution will be reduced to 1/32 of the original size for each image. Due to the small size of mosquitoes, they occupy fewer pixels in the entire image. Too many pooling layers will cause serious loss of mosquitoes information, resulting in a low-quality density map and large counting error. Besides too deep FEN will increase the number of model parameters, making the training time be longer. We use the first ten layers of VGG-16 model as the FEN to extract low-level features of mosquitoes. The FEN uses the three max-pooling layers of VGG-16 network so that the output feature map is reduced to 1/8 of the original size for each image, which can reduce the number of parameters and speed up training while ensuring good performance. And max-pooling layer helps to preserve the texture information of the image, which makes the model focus on the texture features of mosquitoes as much as possible.

### Attention based multi-scale regression network (AMRN)

The AMRN further extracts more complex high-level features of different sizes and estimates pixel-level density map by a regression network. Convolution kernels of the same size receptive field are unlikely to capture features of mosquitoes with different scales^17^. Inspired by the literature^17,23^, we proposed a multi-scale feature fused network using convolution kernels of the local receptive field with different sizes to achieve the feature maps of mosquitoes with different scales. The AMRN has three different sizes of receptive fields to extract features. It consists of three parallel CNNs with the convolution kernel sizes $3\times 3$, $5\times 5$, and $7\times 7$, and each CNN has 3 convolution layers with successively decreasing number of channels, as shown in Fig. 1. For large scale mosquitoes, the convolution kernel with a small-sized receptive field is easy to extract their features, but it is necessary to increase the size of the receptive field of the convolution kernel to enhance its adaptability to small scale mosquitoes. It is natural to use convolution kernels with different sizes that can be sensitive to mosquito features of different scales. Considering the inadequate fitting ability of the linear model, we adopted ReLU as the activation function. We concatenated output feature maps of all CNNs and then map them into a density map using a 1 × 1 convolution kernel.

**Figure 2:**
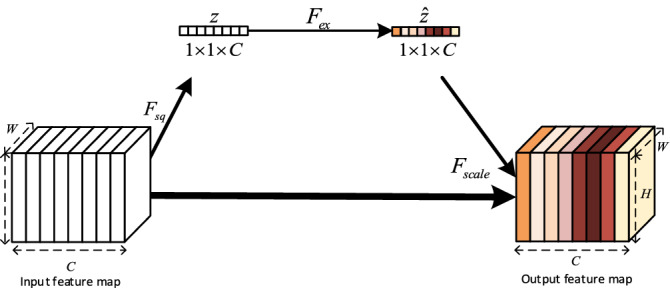
The structure of SE attention module. The squeeze operation is performed to compress a feature map with size $\mathrm{H}\times \mathrm{W}\times \mathrm{C}$ to $1\times 1\times \mathrm{C}$ by using a global average pooling layer (${F}_{sq}$) and get channel-level global features $z$. The excitation operation (${F}_{ex}$) is performed on the feature to obtain the weights of different channels $\widehat{z}$. The final output is obtained by multiplying the original feature map ${F}_{scale}$ with the weights $\widehat{z}$.

Due to different parameters in the convolution kernels, different branches extract different features. We used an SE channel attention module^24^ to increase the sensitivity of the network to the informative features and to suppress the unuseful ones. SE attention module contains SE operations as shown in Fig. 2. Considering the fact that features of some channels are not helpful for mosquito swarm counting because the missing features of the input image after 3 max-pooling layers in the FEN make it difficult to extract the high-level features of mosquitoes, especially in small scale mosquitoes images, we introduced the SE channel attention module in the branch with the convolution kernel size $7\times 7$ in the multi-scale network. It increases the weight of the mosquito features channel and pays more attention to mosquito features.

## Results and discussion

To verify the effectiveness of the proposed model, we evaluated experiments on the built mosquito swarm dataset. We conducted comparative experiments on the proposed network with different settings, including the different pre-trained networks in the FEN, different scales in the AMRN, the location of the SE module, and whether SE modules exist or not.

### Mosquito swarm dataset

**Figure 3:**
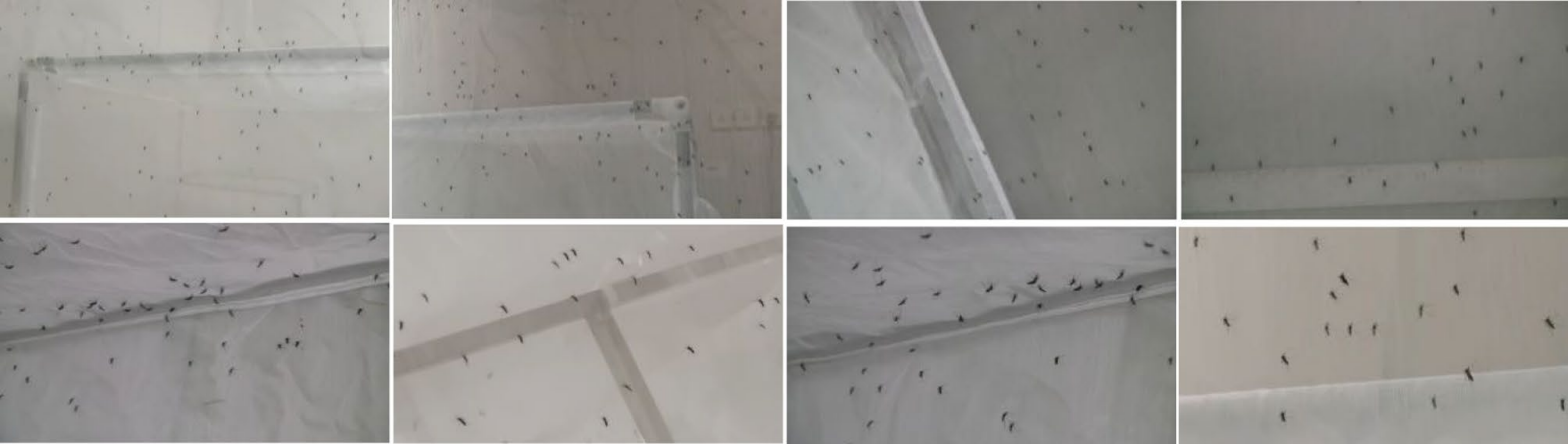
An illustration of mosquito swarm images. The 1st row shows class 1 to 4 from left to right and the 2nd row shows class 5 to 8 from left to right.

The images of mosquito swarm in the data set were collected from the Zhejiang Provincial Center for Disease Control and Prevention. The images were captured and the size of each image is $960\times 540$. The data set is split into the training set and test set, and there are 235 mosquito swarm images in the training set and 156 mosquito swarm images in the test set with 15,466 mosquitoes in total. Images contain mosquitoes with different sizes due to perspective effect. We screened out as many mosquito swarm images with different counts and sizes as possible for training. Some typical images are shown in Fig. 3.

### Ground truth density map of mosquito swarm generation

**Figure 4:**
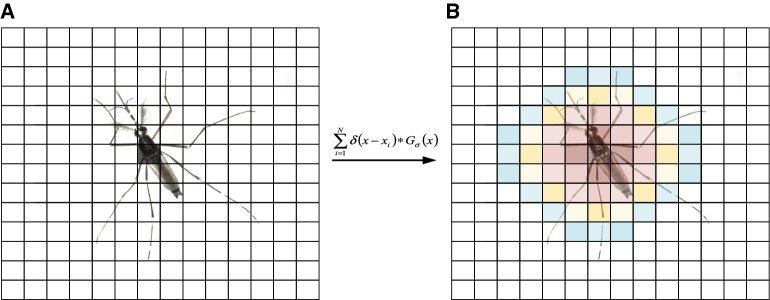
The process of generating one density map. Grids represent pixels. The value of the black pixel in Figure (**A**) represent 1, and the sum of the color pixels in Figure (**B**) is 1.

We used the proposed network to estimate the mosquito swarm density map from an input image, and the ground truth density map quality in the training data largely determines the performance of our method. To effectively extract features of mosquitoes with different scales, we empirically classified the images into 8 classes according to the size of the mosquitoes, as shown in Fig. 3, and their corresponding number of images are shown in Table 1. If there is a mosquito at pixel ${x}_{i}$, we represent it as a delta function $\delta \left(x-{x}_{i}\right)$. So an image with $N$ labelled mosquitoes can be written as $H(x)=\sum_{i=1}^{N}\delta \left(x-{x}_{i}\right)$, where $H(x)$ is built based on the assumption that each pixel ${x}_{i}$ is an independent sample in the image. However, this is not true because different pixels ${x}_{i}$ may correspond to regions with different sizes due to perspective effect. So we convert it to a continuous density function by convolving it with a Gaussian kernel ${G}_{\sigma }$ where $\sigma$ is the size of the Gaussian kernel and is associated with the size of the mosquitoes in the image, and we have the density $F\left(x\right)=\sum_{i=1}^{N}\delta \left(x-{x}_{i}\right)*{G}_{\sigma }\left(x\right)$. And the process of generating one density map is shown in Fig. 4.

**Table 1:** Classes of mosquito swarm images and their corresponding number of images.

Class Index	1	2	3	4	5	6	7	8
Number of images	26	30	73	82	67	33	66	14

**Figure 5:**
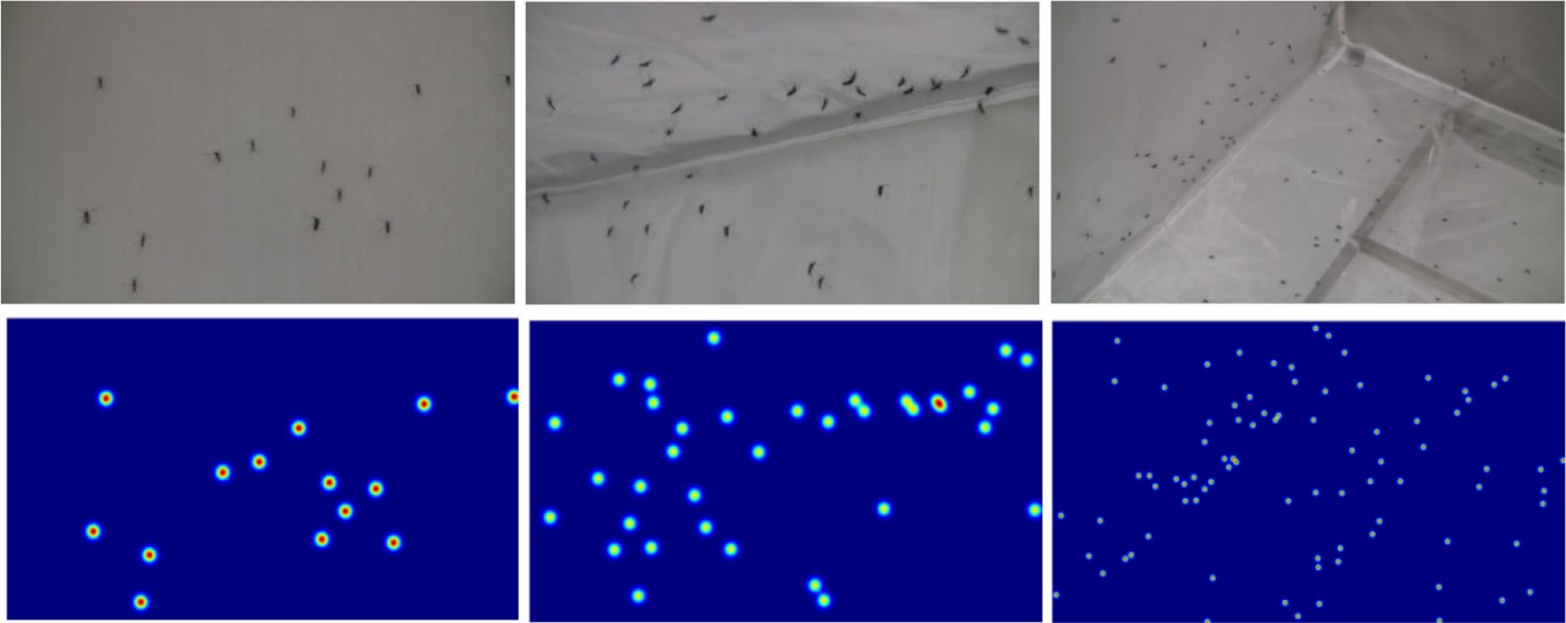
The original images and their corresponding ground-truth density maps. The 1st row shows the original images, and the 2nd row shows the corresponding ground truth density maps of the original images.

Considering the fact that the data set contains mosquitoes of different sizes, we can not accurately get the sizes of mosquitoes to determine the sizes of Gaussian kernels. But we found that it is effective to empirically classify the mosquito sizes into 8 classes with their corresponding ${\sigma }_{i}\in \{\mathrm{2,3},...,9\}$. The procedure for the density map is detailed in Eq. (1). And some of the results are shown in Fig. 5.

1 $$F\left(x\right)=\sum_{i=1}^{N}\delta \left(x-{x}_{i}\right)*{G}_{{\sigma }_{i}}\left(x\right),{\sigma }_{i}\in \left\{2,\cdots ,9\right\}$$

### Loss function

Euclidean distance is used to measure the difference between the estimated density map and the ground truth density map. The loss function is defined as Eq. (2):

2 $$L\left(\Theta \right)=\frac{1}{2N}\sum_{i = 1}^{N}{\Vert P\left({X}_{i};\Theta \right)-{P}_{i}^{GT}\Vert }_{2}^{2}$$

where $\Theta$ is the model parameters of the network. $\mathrm{N}$ is the number of training images. ${X}_{i}$ is the *i*th input image. ${P}_{i}^{GT}$ is the ground truth density map corresponding to the input image ${X}_{i}$, and $P\left({X}_{i};\Theta \right)$ is the estimated density map corresponding to the input image ${X}_{i}$.

### Evaluation metrics

We evaluated the proposed method using mean absolute error (MAE) and root mean square error (RMSE). MAE indicates the accuracy of the predicted values, and RMSE indicates the robustness of the predicted values. The smaller of MAE and RMSE, the better the accuracy of the prediction model. MAE and RMSE are defined as Eqs. (3) and (4) respectively.

3 $$MAE=\frac{1}{N}\sum_{i=1}^{N}\left|{Y}_{i}-{Y}_{i}^{GT}\right|,$$

4 $$RMSE=\sqrt{\frac{1}{N}\sum_{i=1}^{N}{\left|{Y}_{i}-{Y}_{i}^{GT}\right|}^{2}},$$

   Where $N$ is the number of input images, and ${Y}_{i}$ indicates the number of mosquitoes predicted by the *i*th image. ${Y}_{i}$ is written as Eq. (5), where $L$ and $W$ are the height and width of the input image, and ${p}_{l,w}^{i}$ is the pixel value of the predicted density map at $(l, w)$ using the proposed model. ${Y}_{i}^{GT}$ represents the number of mosquitoes in the ground truth density map corresponding to the *i*th image.

5 $${Y}_{i}=\sum_{l=1}^{L}\sum_{w=1}^{W}{p}_{l,w}^{i}$$

### Parameter setting

All experiments are performed on a computer with NVIDIA 1060 GPU, 16 GB RAM running Linux operating system under Anaconda Python3 environment. The back propagation time and stochastic gradient descent (SGD) are adopted for network training. The batch size is 2. The learning rate (LR) is ${10}^{-7}$. The momentum is 0.95, and the weight decay is ${5\times 10}^{-7}$.

## Results

### Performance on mosquito swarm dataset

**Figure 6:**
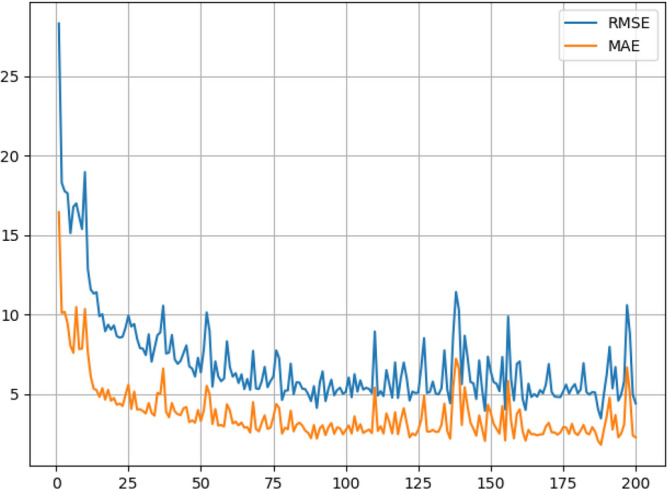
MAE and RMSE of the network in the test dataset. The abscissa is the number of training epochs, and the ordinate is the value of MAE and RMSE.

In the process of network training, MAE and RMSE in the test dataset are measured after each epoch. We showed the curves for MAE and RMSE in Fig. 6. They show an oscillatory downward trend. Although the oscillations are violent in some places, MAE converges to about 2 and RMSE to about 4.

**Figure 7:**
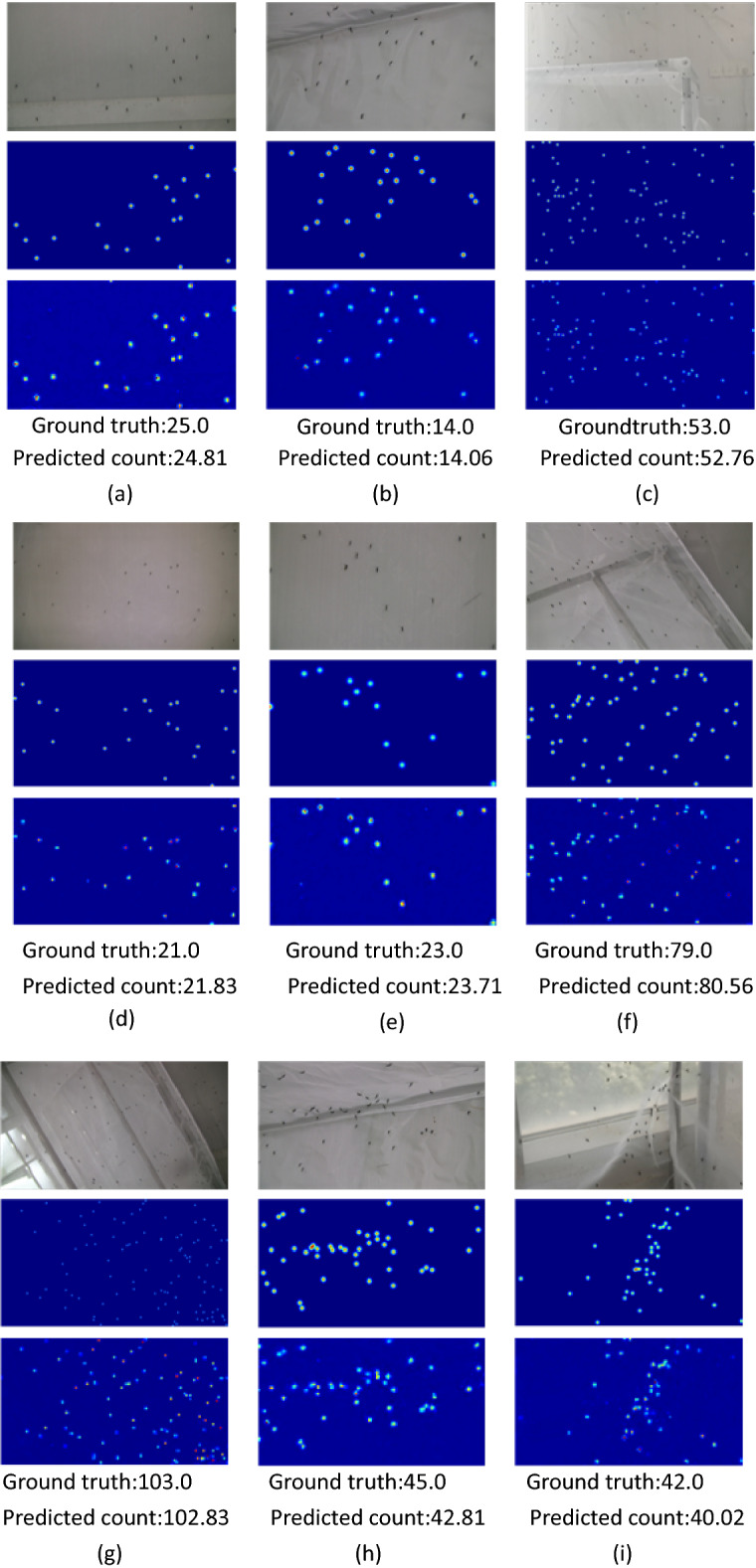
The estimated density maps and mosquito swarm counts. Group (**a**) to (**e**) indicate the results of the images of normal mosquito swarm, and group (**f**) to (**i**) indicate that the mosquito swarm is dense in the images or adhesion exists between the mosquitoes. Each group consists of three rows. The 1st row shows the original images in the test set. The 2nd row shows the ground truth for each original image while the 3rd row shows the estimated density maps by the proposed network.

We tested the network on the test dataset, and achieved the best result with MAE of 1.810 and RMSE of 3.467. Figure 7 shows some examples. Ground truth value is the mosquito swarm count, and the predicted count is the estimated value of the proposed model. The results show that the model presents a more pleasing counting ability for complex situations, such as different densities of mosquito swarm and adhesion between the mosquitoes.

## Discussion

### Ablation on the proposed network structure

We compared the results of different pre-trained networks in the FEN. VGG-16, ResNet-18 and InceptionV3 are the three common pretrained networks. We tried to deploy them as the FEN to extract low-level features in the proposed network respectively, and their results are shown in Table 2. From Table 2, we can see VGG-16 has the best result, so we choose VGG-16 as the FEN in our network.

**Table 2:** MAE and RMSE of the different pre-trained networks in the FEN.

Metrics	VGG-16	ResNet-18	InceptionV3
MAE	**3.259**	5.346	9.612
RMSE	**5.547**	9.570	12.949

Significant values are in bold.

We also compared the results of different numbers of convolution Layers in the FEN. The backbone of the FEN is VGG-16 network. We conducted experiments on the proposed network on the training set with different numbers of convolution layers in the VGG-16 network, and their corresponding MAE and RMSE are shown in Table 3. In Table 3, the 10-layer FEN has the best result. The 7-layer FEN has too few convolutional layers for mosquito swarm images, which limits its representation ability of mosquito features due to its relatively simpler features. The 13-layer FEN has 5 max-pooling layers, which causes excessive features loss and results in poor results.

**Table 3:** MAE and RMSE of the different numbers of convolution layers in the FEN.

Metrics	7 layers	10 layers	13 layers
MAE	6.328	**3.259**	3.559
RMSE	9.685	**5.547**	6.232

Significant values are in bold.

We also compared the results on the single-scale and multi-scale AMRN. And Table 4 lists their corresponding MAE and RMSE. It can be seen from Table 4 that the multi-scale model is much better than the single-scale model about 25% in MAE, and is slightly inferior to the single-scale model about 4.5% in RMSE. From the comparison, we know that the performance of the multi-scale model is better than that of the single-scale model. We used a multi-scale network structure to implement the AMRN since convolution kernels of different sizes have different sensitivity to mosquitoes with different scales.

**Table 4:** MAE and RMSE of the AMRN with different scales.

Metrics	Single-scale	Multi-scale
MAE	2.875	**2.145**
RMSE	**4.139**	4.327

Significant values are in bold.

In order to study the influence of the SE channel attention module in the model, we evaluated its experimental results on three branches 3 × 3, 5 × 5, and 7 × 7 respectively. And the experimental results are shown in Table 5. In Table 5, the numbers in parentheses from column 3 to 5 indicate the convolution kernel size of the branch where the SE module lies. For completeness of the comparison, we also compared the results of three branches with or without SE channel attention modules. It can be seen from the results that the case that the SE channel attention module is on the 7 × 7 branch is better than others in MAE and RMSE. This verifies that the SE channel attention module can further improve sensitivity to small size mosquitoes. But when SE channel attention modules are introduced in each branch, the performance of the model deteriorates.

**Table 5:** Influence of SE attention module in the multi-scale regression network.

Metrics	No attention module	Attention module(3)	Attention module(5)	Attention module(7)	All attention modules
MAE	2.145	2.016	2.116	**1.810**	2.353
RMSE	4.327	3.847	4.392	**3.467**	4.926

Significant values are in bold.

### Performance comparison with other methods

To highlight the performance of the proposed model, we compared the proposed method with the MCNN method^17^ and the CSRNet method^18^ on the complete test set. These two methods are classical density map based regression methods in the field of crowd counting. MCNN model contains three parallel CNNs and each parallel includes four columns CNNs. Different parallel CNNs have different sizes for modeling the density maps corresponding to objects of different scales. MCNN stacks the output of all CNNs and maps them to a density map. CSRNet method uses the first 10 layers of VGG-16 as the front-end network and dilated convolutional layers as the back-end network to enlarge receptive fields and extract deeper features. Compared with the other two methods, the performances of MCNN and CSRNet method are inferior to our method. Unlike the proposed network, MCNN model lacks of an FEN and different parallel CNNs have the same architectures except for the sizes and numbers of filters. So, on the one hand, our network is a deeper network, which can extract more complex features than MCNN. On the other hand, we introduced the SE module that boosts informative features of the mosquitoes, into the regression network to increase the asymmetry of the network structure, so that the network can learn more different features than MCNN. For CSRNet, it can extract deeper features and obtain smaller MAE than the MCNN, but it is the network with sing-scale and could not adapt to mosquitoes of different sizes. Meanwhile, the introduction of SE module in our regression network makes CSRNet further disadvantaged. The experimental results are shown in Table 6, and we can see that our method is superior to CSRNet and MCNN. Our method has the smallest MAE and RMSE.

**Table 6:** Comparison between our method and other two methods.

Metrics	CSRNet^18^	MCNN^17^	Our method
MAE	4.225	5.266	**1.810**
RMSE	7.796	7.721	**3.467**

Significant values are in bold.

## Conclusion

We have successfully applied the deep learning and channel attention module in mosquito swarm counting. We first used the modified VGG-16 network to build the feature extraction network to extract low-level features of mosquitoes, then adopted a multi-scale regression network with SE channel attention module to extract more complex high-level features and mapped feature maps to mosquito swarm density maps. To better evaluate the performance of the proposed method, we collected and labelled a data set including about 391 mosquito swarm images with 14,566 mosquitoes. Our method performs well on the data set and achieves mean absolute error (MAE) of 1.810 and root mean square error (RMSE) of 3.467. In the future work, we will study mosquito swarm counting methods applicable to mobile terminals, especially the deep learning model based on lightweight network.

## Data Availability

Source data are available from Huahua Chen (iseealv@hdu.edu.cn) upon request.

## Code availability

All code related to the model was implemented in Python. Code related to the deep learning models is available from the authors upon request.
